# Diagnostic testing practices for diarrhoeal cases in South African public hospitals

**DOI:** 10.1186/s12879-022-07834-0

**Published:** 2022-11-09

**Authors:** Siobhan L. Johnstone, Nicola A. Page, Michelle J. Groome, Nicolette M. du Plessis, Juno Thomas

**Affiliations:** 1 Centre for Enteric Diseases, National Institute for Communicable Diseases, a division of the National Health Laboratory Service (NHLS), Johannesburg, South Africa; 2 School of Public Health, Faculty of Health Sciences, University of the Witwatersrand, Johannesburg, South Africa; 3 Department of Medical Virology, Faculty of Health Sciences, University of Pretoria, Private Bag X323, Arcadia, 0007 South Africa; 4 Division of Public Health Surveillance and Response, National Institute for Communicable Diseases, a division of the National Health Laboratory Service (NHLS), Johannesburg, South Africa; 5 School of Pathology, Faculty of Health Sciences, University of the Witwatersrand, Johannesburg, South Africa; 6 Department of Paediatrics, Kalafong Provincial Tertiary Hospital, Faculty of Health Sciences, University of Pretoria, Pretoria, 0001 South Africa

**Keywords:** Diarrhoea, Diagnostics, Aetiology, Low resource, Stool samples, Surveillance

## Abstract

**Background:**

Stool samples submitted for diagnostic testing represent a proportion of diarrhoeal cases seeking healthcare, and an even smaller proportion of diarrhoeal cases in the community. Despite this, surveillance relies heavily on these laboratory results. This study described diarrhoeal diagnostic practices and aetiological agents of diarrhoea in patients admitted to three South African public hospitals in order to understand biases in surveillance data, and inform guidelines, diagnostic and laboratory practices to improve clinical management.

**Methods:**

A doctors’ survey was conducted to determine sample submission, diarrhoeal treatment and barriers to submitting samples for testing. Results for all samples submitted for routine diagnostics were obtained from the NHLS Central Data Warehouse. An enhanced surveillance study enrolled patients with acute diarrhoea at the same hospitals over the same period. Differences between routine culture results and molecular testing from the surveillance study were described.

**Results:**

Stool samples were seldom submitted for diagnostic testing (median of 10% of admitted cases). Current diagnostic guidelines were not useful, hence most doctors (75.1%) relied on their own clinical judgement or judgement of a senior clinician. Although most doctors (90.3%) agreed that diagnostics were helpful for clinical management, they reported patients being unwilling to provide samples and long laboratory turnaround times. Routine diagnostic data represent cases with chronic diarrhoea and dysentery since doctors are most likely to submit specimens for these cases. Pathogen yield (number of pathogens detected for samples tested for specific pathogens) was significantly higher in the surveillance study, which used molecular methods, than through routine diagnostic services (73.3% versus 8.2%, p < 0.001), including for viruses (48.9% versus 2.6%, p < 0.001), bacteria (40.1% versus 2.2%, p < 0.001) and parasites (16.2% versus 3.6%, p < 0.001). Despite viruses being commonly detected in the surveillance study, viral testing was seldom requested in routine diagnostic investigations.

**Conclusions:**

Comprehensive diagnostic and treatment guidelines are required for diarrhoeal diseases. These guidelines should be informed by local epidemiological data, where diagnostic testing is reserved for cases most likely to benefit from specific treatment. Optimisation of current diagnostic processes and methods are required for these cases, specifically in terms of minimising turnaround times while maximising diagnostic acumen.

## Introduction

Diarrhoeal surveillance relies primarily on routine diagnostic laboratory data, even in settings with established surveillance systems. Analysis of routine diagnostic data is useful in describing trends for enteric pathogens, but such data represent a minor subset of diarrhoeal cases in the community. Only those cases consulting a healthcare professional and for whom the healthcare professional orders laboratory screening of a stool sample are included in this subset. In South Africa, the relevance of routine diagnostic data is not clear, since little is known about the factors influencing healthcare professionals’ diagnostic choices at different healthcare levels. An understanding of diagnostic testing practices is important for the interpretation of routine diagnostic and laboratory-based surveillance data, specifically when determining how much these data underestimate the true burden of disease [1]. Knowledge of factors that influence doctors’ diagnostic practices is required, not only to understand the strengths and weaknesses of routine diagnostic data [2], but also to highlight gaps in routine diagnostic services and guidelines [3] for improving patient management.

Diarrhoeal diagnostics should be limited to cases in which clinical management may benefit from knowledge of the aetiological agent, and tests should be limited to pathogens likely to be present in the population [3]. Guidelines should hence be based on local or regional epidemiological data, minimizing costs for healthcare and patients [3]. South African National Department of Health guidelines for the treatment of diarrhoea are included in the standard treatment guidelines and essential medicines list (hospital and primary healthcare levels) [4, 5]. An audit at a provincial hospital in KwaZulu-Natal found that patients with diarrhoea were managed inconsistently, specifically with regards to antibiotic treatment, which could result in an increased risk of antibiotic resistance and infection with *Clostridioides difficile (C. difficile)* [6]. The study reported that stool samples were submitted for routine microscopy (examination for ova and parasites) and culture in 47% of cases, yet 60% of cases were treated with antibiotics (only 35% of which had positive culture results). The authors called for clearer national guidelines for the management of diarrhoea, particularly for HIV-infected patients who comprised the majority (81%) of diarrhoeal cases presenting to the emergency department [6]. Robust epidemiological and aetiological data are needed to formulate nationally relevant guidelines.

When guidelines serve an advisory purpose and are not strictly prescriptive, they are not reliable predictors of diagnostic practice, and it is important to investigate other predictors. Studies in high-income countries have found dysentery [1, 2, 7], a diagnosis of HIV or immunosuppression from other causes [2, 7] and prolonged illness [1, 2, 7] to be strong predictors for requesting stool culture. In these settings, routine diagnostic data are likely to underestimate the pathogens that rarely cause dysentery while pathogens such as *Escherichia coli* 0157:H7 are likely to be overrepresented [2]. A strong association between requesting stool culture and the need for hospitalization, intravenous rehydration, further consultation and a diagnosis of HIV, means that surveillance data are biased towards patients who present with severe illness [2]. Other reported predictors include overseas travel and suspected links to an outbreak [1]. The rates of requests for stool culture differ between age groups, with studies from high-income settings indicating increased sample submissions for the young (< 5 years) and the elderly (≥ 60 years) [8], influencing the interpretation of surveillance data. The availability of laboratory services, pressure from laboratories to limit number of samples submitted, availability of sample collection kits, cost of tests, confidence in laboratory results, and turn-around-time for laboratory results are reported barriers preventing laboratory screening [7].

The aims of this study were to describe the diagnostic practice for diarrhoea at three hospitals in South Africa; to identify what informs these decisions; to understand clinicians’ perceptions regarding laboratory tests; and to describe the aetiological agents of diarrhoea in patients admitted to these hospitals. The results will elucidate reasons for the bias in surveillance data, and inform recommendations for improving the management of diarrhoea in terms of guidelines, diagnostic practice and laboratory testing.

## Methods

### Study sites

The study was conducted at three hospitals (Kalafong, a provincial, tertiary hospital in an urban area in Gauteng Province; Matikwana, a regional hospital, and Mapulaneng, a district hospital, both in rural Mpumalanga Province) which are study sites for the African Network for Improved Diagnostics, Epidemiology and Management of common Infectious Agents (ANDEMIA), details of which have been described [9].

### Attending doctors’ survey

A survey was conducted among doctors attending patients in all wards (including paediatrics, adult medical, surgical, emergency, obstetrics and outpatient wards) at the three surveillance hospitals in October–November 2020. The questionnaire consisted of sections on general information (demographics, position in the hospital, speciality and ward); stool sample submission and diarrhoeal treatment practices; and barriers to submitting stool samples for testing. Virology, epidemiology and clinical experts revised the questionnaire before implementation. The survey was briefly presented to the doctors at departmental meetings and hard copy questionnaires completed during the meetings. An electronic version was emailed to those not present at the meetings using a web-based platform (Castor EDC). Questionnaires were captured onto an electronic database.

### Surveillance study

Prospective, hospital based, surveillance was conducted at the same study hospitals over an 18-month period (July 2018 to December 2019). Surveillance officers based at the hospitals enrolled patients of all ages presenting with acute diarrhoea (defined as three or more loose stools per day for 28 days or less). The study was explained to patients and those consenting were requested to provide a stool sample or rectal swab. Surveillance officers conducted interviews to determine demographic variables and risk factors. Stool samples were sent to the Centre for Enteric Diseases at the National Institute for Communicable Diseases (NICD) where real-time polymerase chain reaction (PCR) testing was performed using Fast Track Diagnostics assays for common viral, bacterial and parasitic causes of gastroenteritis. Detailed study methods are available [9].

### Routine diagnostic test data

The tests routinely ordered for stool microbiology in the public health sector is colloquially termed stool ‘MCS’, and refers to stool microscopy for ova and parasites, culture, and antimicrobial susceptibility testing where applicable. Results for all stool and rectal swab samples submitted for routine microscopy for ova and parasites and culture as well as any additional microbiologic or virologic testing, to diagnostic laboratories at the study hospitals for the period July 2018 to December 2019 were extracted from the Central Data Warehouse (CDW) of the National Health Laboratory Services (NHLS). Data were de-identified with only patient sex and age included. Submission of samples for routine diagnostic testing by attending doctors was independent of enrolment in the surveillance study. The denominator for pathogen yield was the number of samples tested for a specific pathogen (as opposed to total samples collected since not all samples were tested for all pathogens).

### Statistical analysis

Characteristics of survey respondents were described using proportions, medians and interquartile ranges (IQR). Responses for guidance of decision-making, factors associated with and barriers to sample submission were compared between urban and rural sites using Chi-squared test and Fisher’s two-tailed exact test, as appropriate.

CDW data were used to determine proportions of submitted samples tested for specific pathogens. CDW data and surveillance study data for the same period were compared using Chi-squared/Fisher’s two-tailed exact tests. Significant results were defined by p ≤ 0.05. All data analysis was conducted using Stata software (version 14).

### Ethics

The ANDEMIA surveillance study was approved by the Human Research Ethics Committee (Medical) at the University of the Witwatersrand (Approval number: M170403) and the University of Pretoria (Approval number: 101/2017). The doctors’ survey and analysis of CDW data was approved by the Human Research Ethics Committee (Medical) at the University of the Witwatersrand (Approval number: M190663 MED18-12-034). Permission for the use of CDW data was obtained from the NHLS.

## Results

### Doctors’ survey respondents

There were 32 responses to the doctors’ survey, included medical officers (n = 17), interns (n = 8), registrars (n = 5), and specialists (n = 2). Respondents were based in paediatric (n = 14), outpatient (n = 8), obstetrics and gynaecology (n = 6), adult medical (n = 6), emergency medicine (n = 2) and surgical (n = 2) departments. The majority (67.7%) of respondents attended to ≤ 5 cases of diarrhoea per week, with a median of four cases per week (IQR 2–8 cases). Respondents at the urban site attended more cases per week than the rural sites (5.5 versus 3) although this difference was not significant (p = 0.095). The percentage of diarrhoeal cases for which a stool sample was requested varied widely between respondents (range 0–100%, median of 10% and IQR of 10–70%). Respondents at the urban site reported ordering stool screening for a higher number of cases than those at the rural sites (42.5% versus 10%), although this difference was not significant (p = 0.054).

### Guidance for decision-making

There was poor agreement among the respondents as to how decisions for ordering stool testing, prescribing antibiotic therapy and the treatment of diarrhoea are governed (Table 1). The majority of respondents did not follow any guidelines for ordering stool tests, relying rather on their own clinical judgement (43.8%) or the guidance of senior clinicians (31.3%). This finding was consistent at both urban and rural sites. The majority of respondents followed treatment guidelines for the prescription of antibiotics (46.9% followed national guidelines and 15.6% followed hospital guidelines). Doctors at the rural sites were more likely to follow national guidelines than those at the urban site (59.1% versus 20.0%, p = 0.060), although this was not significant. National guidelines were often followed for treatment of diarrhoeal cases (59.4%), specifically for the rural as compared to the rural sites (72.7% versus 30.0%, p = 0.049).

**Table 1:** Guidance for decision-making

	Total (n = 32)	Urban (n = 10)	Rural (n = 22)	p-value
Stool sample submission practices^a^				
National guidelines	9 (28.1%)	1 (10. 0%)	8 (36.4%)	0.210
Hospital policy	4 (12.5%)	3 (30.0%)	1 (4.6%)	0.079
Senior clinician guidance	10 (31.3%)	5 (50.0%)	5 (22.7%)	0.217
Own clinical judgement	14 (43.8%)	5 (50.0%)	9 (40.9%)	0.459
Antibiotic prescription practices^a^				
National guidelines	15 (46.9%)	2 (20.0%)	13 (59.1%)	0.060
Hospital policy	5 (15.6%)	1 (10.0%)	4 (18.2%)	> 0.999
Senior clinician guidance	8 (25.0%)	4 (40.0%)	4 (18.2%)	0.218
Own clinical judgement	8 (25.0%)	4 (40.0%)	4 (18.2%)	0.218
Treatment of diarrhoeal cases^a^				
National guidelines	19 (59.4%)	3 (30.0%)	16 (72.7%)	**0.049**
Hospital policy	3 (9.4%)	0 (0.0%)	3 (13.6%)	0.310
Senior clinician guidance	10 (31.3%)	4 (40.0%)	6 (27.3%)	0.683
Own clinical judgement	7 (21.9%)	4 (40.0%)	3 (13.6%)	0.165

*P*-values < 0.05 indicated in bold

^a^More than one answer could have been selected per respondent hence the total may exceed 100%

### Patient-related factors associated with ordering stool tests

Respondents were likely to order stool tests for patients with chronic diarrhoea (81.3% of respondents would submit a sample), dysentery (65.6%), suspected *C. difficile* infection (62.5%), travel history (53.1%) or suspected link to an outbreak (53.1%) (Table 2). Patient-related factors associated with ordering stool screening were similar between urban and rural sites, except for HIV-related diarrhoea which was more likely to be associated with having a stool sample ordered at the urban site (70.0% versus 27.3%, p = 0.049).

**Table 2:** Patient-related factors influencing the likelihood of a doctor ordering stool tests

	Total (n = 32)	Urban (n = 10)	Rural (n = 22)	p-value
Case characteristic				
Chronic diarrhoea	26 (81.3%)	9 (90.0%)	17 (77.3%)	0.637
Dysentery	21 (65.6%)	6 (60.0%)	15 (68.2%)	0.703
Suspected *C. difficile* infection	20 (62.5%)	8 (80.0%)	12 (54.6%)	0.248
History of travel	17 (53.1%)	6 (60.0%)	11 (50.0%)	0.712
Suspected link to an outbreak	17 (53.1%)	6 (60.0%)	11 (50.0%)	0.712
Known HIV infection	13 (40.6%)	7 (70.0%)	6 (27.3%)	**0.049**
Suspected foodborne illness	12 (37.5%)	4 (40.0%)	8 (36.4%)	> 0.999
Suspected healthcare-associated infection	12 (37.5%)	3 (30.0%)	9 (40.9%)	0.703
Children under 5 years of age	11 (34.4%)	2 (20.0%)	9 (40.9%)	0.425
Suspected rotavirus infection (during rotavirus season)	7 (21.9%)	1 (10.0%)	6 (27.3%)	0.387
Adults ≥ 65 years of age	5 (15.6%)	2 (20.0%)	3 (13.6%)	0.506
Fever	7 (21.9%)	3 (30.0%)	4 (18.2%)	0.648
Dehydration requiring intravenous fluid replacement	6 (18.8%)	1 (10.0%)	5 (22.7%)	0.637
Vomiting	2 (6.3%)	0 (0.0%)	2 (9.1%)	> 0.999

*P*-values < 0.05 indicated in bold

### Barriers to ordering stool tests

Most respondents (90.3%) reported that stool diagnostics assisted with the clinical management of diarrhoea (Table 3). More than half of the respondents (58.6%) indicated that they would order stool diagnostics more often in the absence of barriers, specifically at the rural sites (70.0% versus 33.3%, p = 0.064). The most frequently reported barriers to ordering stool tests were laboratory turnaround times being too long to assist with clinical decision-making (reported by 54.8% of respondents) and patients being unwilling to provide stool samples (35.5%). Financial constraints on testing was a barrier reported by 23.8% of respondents at the rural sites, but by none at the urban site (p = 0.092). More respondents at the urban site reported a lack of confidence in laboratory results than the rural sites (40.0% versus 9.5%, p = 0.067). Other barriers reported by respondents included high patient numbers, the need to reserve testing for cases not responding to supportive treatment, and challenges with sample labelling, and transport to the laboratory.

**Table 3:** Reported barriers to ordering stool tests

	Total (n = 32)	Urban (n = 10)	Rural (n = 22)	p-value
Laboratory turnaround times too long to assist with clinical decision-making	17 (54.8%)	6 (60.0%)	11 (52.4%)	> 0.999
Patients unwilling to provide stool samples	11 (35.5%)	3 (30.0%)	8 (38.1%)	0.660
Nursing staff reluctant to collect stool samples	10 (32.3%)	5 (50.0%)	5 (23.8%)	0.222
Lack of confidence in laboratory results	6 (19.4%)	4 (40.0%)	2 (9.5%)	0.067
Financial constraints on diagnostic testing	5 (16.1%)	0 (0.0%)	5 (23.8%)	0.092
Diagnostic results are unhelpful in clinical management/decision making	3 (9.7%)	1 (10.0%)	2 (9.5%)	> 0.999

### Pathogens tested for in routine stool diagnostic tests

The majority of doctors believed that *Salmonella* spp. (84.4%), *Shigella* spp. (84.4%) and enterohaemorrhagic *Escherichia coli* (EHEC) (53.1%) were included in routine stool MCS (Table 4). Many respondents also thought that *Giardia lamblia* (50.0%), rotavirus (37.5%), adenovirus (37.5%) and norovirus (15.6%) were tested for in routine stool MCS.

**Table 4:** Pathogens doctors believed to be included in routine stool MCS investigations

	Total (n = 32)	Urban site (n = 10)	Rural sites (n = 22)	p-value
Bacteria				
*Salmonella* spp.	27 (84.4%)	10 (100.0%)	17 (77.3%)	0.155
*Shigella* spp.	27 (84.4%)	10 (100.0%)	17 (77.3%)	0.155
EHEC	17 (53.1%)	4 (40.0%)	13 (59.1%)	0.316
*Campylobacter* spp.	13 (40.6%)	4 (40.0%)	9 (40.9%)	> 0.999
All Shiga toxin-producing *E. coli* (STEC)	13 (40.6%)	5 (50.0%)	8 (36.4%)	0.699
*C. difficile*	11 (34.4%)	4 (40.0%)	7 (31.8%)	0.703
All diarrhoeagenic *E. coli*	9 (28.1%)	4 (40.0%)	5 (22.7%)	0.407
*Vibrio* spp.	4 (12.5%)	0 (0.0%)	4 (18.2%)	0.283
*Yersinia enterocolitica*	3 (9.4%)	1 (10.0%)	2 (9.1%)	> 0.999
Parasites				
*Giardia lamblia*	16 (50.0%)	4 (40.0%)	12 (54.6%)	0.704
*Cryptosporidium* spp.	10 (31.3%)	3 (30.0%)	7 (31.8%)	< 0.999
*Cystoisospora* spp.	9 (28.1%)	3 (30.0%)	6 (27.3%)	< 0.999
*Entamoeba histolytica*	9 (28.1%)	2 (20.0%)	7 (31.8%)	0.681
*Microsporidia* spp.	5 (15.6%)	2 (20.0%)	3 (13.6%)	0.637
*Cyclospora* spp.	2 (6.3%)	2 (20.0%)	0 (0.0%)	0.091
*Aeromonas* spp.	0 (0.0%)	0 (0.0%)	0 (0.0%)	–
Viruses				
Rotavirus	12 (37.5%)	2 (20.0%)	10 (45.5%)	0.248
Adenovirus	12 (37.5%)	2 (20.0%)	10 (45.5%)	0.248
Norovirus	5 (15.6%)	1 (10.0%)	4 (18.2%)	> 0.999

‘MCS’ refers to microscopy for ova and parasites, culture, and antimicrobial susceptibility testing where applicable

CDW data indicate that most stool samples at both rural and urban sites were tested for *Salmonella* spp. (99.9%) and *Shigella* spp. (99.8%) (Table 5). Microscopy for ova and parasites was frequently included at rural and urban sites (86.5% and 92.7% respectively). Routine testing at the urban site frequently included culture for *Campylobacter* spp. (71.4%) and EHEC (55.3%) but culture for these pathogens was rarely performed at the rural sites (1.1% and 1.1% respectively). Testing for viruses was infrequent as specific requests are required for testing (rotavirus tested for in 16.8%, adenovirus in 15.7% and astrovirus in 3.1% of samples) and was exclusive to the urban site. Culture for *Vibrio cholerae* was done for the majority of samples at the rural sites (96.6%), but not for any samples from the urban site. There was a median of 4.6 days (IQR: 3.5–6.2) between sample collection and reporting of results for routine stool MCS.

**Table 5:** Pathogens tested for in stool samples submitted to diagnostic laboratories

	Total (n = 1 311)	Urban site (n = 1 222)	Rural sites (n = 89)	p-value
*Salmonella* spp.	1309 (99.9%)	1220 (99.8%)	89 (100.0%)	0.702
*Shigella* spp.	1308 (99.8%)	1219 (99.8%)	89 (100.0%)	0.640
Parasites	1210 (92.3%)	1133 (92.7%)	77 (86.5%)	**0.034**
*Campylobacter* spp.	873 (66.6%)	872 (71.4%)	1 (1.1%)	**< 0.001**
EHEC	677 (51.6%)	676 (55.3%)	1 (1.1%)	**< 0.001**
*C. difficile*	375 (28.6%)	374 (30.6%)	1 (1.1%)	**< 0.001**
Rotavirus	220 (16.8%)	220 (18.0%)	0 (0.0%)	**< 0.001**
Adenovirus	206 (15.7%)	206 (16.9%)	0 (0.0%)	**< 0.001**
*Vibrio cholerae*	93 (7.1%)	7 (0.6%)	86 (96.6%)	**< 0.001**
*Vibrio* spp.	90 (6.9%)	6 (0.5%)	84 (94.4%)	**< 0.001**
EPEC	64 (4.9%)	64 (5.2%)	0 (0.0%)	**0.027**
Astrovirus	40 (3.1%)	40 (3.3%)	0 (0.0%)	0.083
Norovirus	0 (0.00%)	0 (0.0%)	0 (0.0%)	–

*P*-values < 0.05 indicated in bold

### Comparison of routine diagnostic data and surveillance study data

Over the study period, 591 stool samples were tested through the surveillance study and 1311 through routine diagnostic services (Table 6). The surveillance study included only patients admitted during regular working hours (Monday to Friday) who consented to study procedures, used a strict case definition of diarrhoea, and required samples to be collected within 48 h of admission to exclude possible healthcare-associated infections. Routine diagnostic services included patients seen in outpatient clinics and in casualty, accounting for the higher number of samples, specifically for the urban site, which has large outpatient clinics. Patients with stool samples submitted through routine diagnostic services were significantly older than those enrolled through the surveillance study (median of 34.0 years versus 1.0 year, p < 0.001), indicating doctors are less likely to request stool testing for admitted children. Proportionally more males were enrolled in the surveillance study compared to females (44.7% versus 50.8%, p = 0.016).

**Table 6:** Comparison of samples submitted for routine diagnostic testing and samples tested through the surveillance study

	Routine diagnostic data (n = 1311)	Surveillance study (n = 591)	p-value
Samples per site			
Kalafong	1222 (93.2%)	311 (52.6%)	< 0.001
Mapulaneng	68 (5.2%)	103 (17.4%)	
Matikwana	21 (1.6%)	177 (29.9%)	
Age groups (years)^a^			
< 1	161 (13.0%)	211 (35.7%)	**< 0.001**
1–4	142 (11.5%)	183 (31.0%)	
5–17	69 (5.6%)	24 (4.1%)	
18–44	540 (43.6%)	98 (16.6%)	
45+	328 (26.5%)	75 (12.7%)	
Gender			
Male	581 (44.7%)	298 (50.8%)	**0.016**
Female	719 (55.3%)	289 (49.2%)	
Samples with pathogen detected	179/1311 (13.7%)	433 (73.3%)	**< 0.001**
Virus detected	34/1311 (2.6%)	289 (48.9%)	**< 0.001**
Adenovirus^b^	10/206 (4.9%)	151 (25.6%)	**< 0.001**
Rotavirus^b^	29/220(13.2%)	92 (15.6%)	0.394
Norovirus	NT	84 (14.2%)	–
Astrovirus^b^	2/40 (5.0%)	35 (5.9%)	0.814
Sapovirus	NT	21 (3.6%)	–
Bacteria detected	105/1311 (8.0%)	237 (40.1%)	**< 0.001**
*Shigella* spp.^b^	10/1308 (0.8%)	134 (22.7%)	**< 0.001**
*Campylobacter*^b^	2/873 (0.2%)	70 (11.8%)	**< 0.001**
*Salmonella* spp.^b^	17/1309 (1.3%)	21 (3.6%)	**0.001**
EHEC^b,c^	0/677 (0.0%)	9 (1.5%)	**0.001**
*C. difficile*	76/375 (20.3%)	34 (5.8%)	**< 0.001**
EPEC^b^	0/64 (0.0%)	NT	–
*Vibrio* spp.^b^	0/90 (0.0%)	NT	–
*Vibrio cholerae*^b^	0/93 (0.0%)	NT	–
Parasites detected	47/1311 (3.6%)	96 (16.2%)	**< 0.001**
*Cryptosporidium* spp.^b^	36/1210 (3.0%)	78 (13.2%)	**< 0.001**
*Giardia lamblia*^b^	0/1210 (0.0%)	22 (3.7%)	**< 0.001**
*Cystoisosopora belli*^b^	8/1210 (0.7%)	NT	–
Other^b,d^	3/1210 (0.2%)		–

*NT* not tested, *EHEC* enterohemorrhagic *Escherichia coli*, *EPEC* enteropathogenic *Escherichia coli*

*P*-values < 0.05 indicated in bold

^a^71 CDW cases missing ages

^b^Denominators for CDW data refer to number tested for specific pathogen rather than total samples submitted

^c^Molecular toxin testing done for EHEC in the surveillance study and by culture for CDW data

^d^Other parasites include: *Endolimax nana*, *Schistosoma mansoni*, *Taenia* spp.

Pathogen yield was significantly higher in the surveillance study than through routine diagnostic services (73.3% versus 13.7%, p < 0.001). This was true for viruses (48.9% versus 2.6%, p < 0.001), bacteria (40.1% versus 8.0%, p < 0.001) and parasites (16.2% versus 3.6%, p < 0.001). Of specific concern was the difference in *Shigella* spp. detection rates (22.7% in the surveillance study and only 0.8% from CDW data). Pathogens most commonly detected through routine diagnostic services included rotavirus (13.2%), adenovirus (4.9%) and *Cryptosporidium* (3.0%), while the surveillance study most commonly detected adenovirus (25.6%), *Shigella* spp. (22.7%) and rotavirus (15.6%). Detection rates for rotavirus (15.6% versus 13.2%, p = 0.394) and astrovirus (5.9% versus 5.0%, p = 0.814) were similar between surveillance and CDW data. Antibiotics were prescribed for 386 (65.3%) of patients enrolled in the surveillance study, however, bacterial pathogens were only detected in 153 (39.6%) of these cases. The high yield of *C. difficile* through routine diagnostic testing (20.3% versus 5.8%, p < 0.001) was expected since only cases with clinically suspected *C. difficile* infection would be tested, whereas the surveillance study screened all samples for various enteric pathogens, regardless of suspected diagnosis.

## Discussion

The doctors’ survey indicates that diarrhoeal cases in rural settings are likely to be underrepresented by routine diagnostic data as rural doctors reported attending fewer diarrhoeal cases and submitted stool samples for a smaller proportion of these cases as compared to their urban counterparts. Possible reasons for this include poorer health-seeking behaviour in rural communities [10], a decentralised public sector healthcare system which encourages patients to utilise local health clinics instead of seeking care directly at hospitals, and less budget for laboratory tests. Stool diagnostic practices at the surveyed sites were based on clinical judgement rather than national or facility guidelines. Doctors were most likely to order stool samples for patients with chronic diarrhoea, dysentery, suspected *C. difficile* infection, a travel history or link to a known or suspected outbreak, all patient-related predictors similar to those reported in studies from high-income countries [1, 2, 7]. Doctors reported ordering stool testing more often for adults than for children even though more children are admitted for diarrhoea, which may introduce an age-related bias to routine diagnostic data. Doctors possibly suspect viral infections in children and, the availability of data on diarrhoea in children allows for empiric treatment.

As per studies in high-income settings [11], the majority of respondents reported that stool sample microbiology was useful in managing patients with diarrhoea and would order stool samples more often in the absence of current barriers. The major barrier to ordering samples was laboratory turnaround times being too long to guide clinical decision-making. Routine diagnostic data indicated an average of 4.6 days (IQR: 3.5–6.2) between sample collection and results, by which time most patients have been discharged. Other important barriers included patients being unwilling to provide samples, and reluctance of nursing staff to assist with sample collection. Other studies have identified laboratory turnaround time as a common barrier to ordering stool samples [7], but none have found reluctance of patients to provide samples or reluctance of healthcare workers to assist in sample collection to be important barriers. The resistance to stool sample collection in our setting could be influenced by many factors. Lack of basic knowledge or guidance on collection procedures and lack of equipment for sample collection may hinder compliance and patients seen in outpatient or casualty may be unable to provide stool samples during the consultation. Rectal swabs or wipes should be investigated to address compliance. Swabs are more convenient for both patients and clinicians and demonstrate decreased time to results [12]. Wipes are efficient in the detection of both bacterial and viral pathogens [13, 14], and collection without requiring nursing assistance, may address compliance issues.

Most doctors (84%) knew that *Salmonella* spp. and *Shigella* spp. were tested for in routine stool MCS. *Campylobacter* spp. and EHEC were tested for in 71.4% and 55.3% of samples respectively at the urban site but were seldom tested for at rural sites (1.1% of samples tested for each). Respondents at the urban site were all aware that *Vibrio cholerae* was not tested for in a routine stool MCS. In contrast, most (86.6%) samples submitted for routine stool MCS at the rural sites were tested for *V. cholerae*, but only 18% of respondents were aware of this. Routine testing for *V. cholerae* is common practice in several areas of South Africa which previously experienced cholera outbreaks and maintain a higher awareness for cholera, as is the case with both rural hospitals. Doctors were not always sure when testing for viruses was done. Many respondents (37.5%) believed that rotavirus and adenovirus were included in stool MCS, but ≤ 18% of samples at the urban site and no samples at the rural sites were tested for these viruses. Some respondents (15.6%) believed norovirus was included in routine testing but CDW data indicated testing was not done at any of the three sites. Overall, no samples from the rural sites and relatively few samples from the urban site were tested for viral pathogens, since clinicians should specifically request this testing. The majority of diagnostic laboratories in the public sector do not perform tests for enteric viral pathogens, and in cases where testing has been requested by the clinician, the samples are referred to an academic or reference laboratory able to perform the test. However, such tests are expensive, and transport to the reference laboratory can take several days, resulting in turnaround time being too long to assist with clinical decision-making.

Pathogen yield for stool samples tested at diagnostic laboratories (routine MCS as well as any additional microbiology or virology tests) was 13.7%, significantly lower than for samples collected in the enhanced surveillance study and tested with real-time PCR (73.3%). Likely reasons for the low yield of pathogens in routine diagnostic laboratory data include pre-analytic factors (timing of sample collection, sample quality, sample storage conditions, transport to laboratory, time from receipt to processing at the laboratory) and analytic factors (quality of testing, pathogen(s) tested for, sensitivity of test(s) performed). Routine diagnostic testing relies primarily on microscopy to detect parasites, and on culture to detect a variable range of bacterial pathogens. Microscopy for ova and parasites is labour intensive and highly dependent on the operator’s skill and expertise [15]. Culture methods are known to deliver poor yields, which typically decrease even further in settings of high antibiotic use [15]. The advantages of multiplex PCR tests are well described; several studies have shown high sensitivity and specificity when used in symptomatic patients, and have shorter turnaround times than conventional culture-based tests [15]. Two landmark studies investigating the burden and aetiology of childhood diarrhoea (the Global Enteric Multicenter Study (GEMS) [16] and the Etiology, Risk Factors, and Interactions of Enteric Infections and Malnutrition and the Consequences for Children Health and Development (MAL-ED) [17] study) tested stool samples using conventional microbiologic methods (culture, enzyme immunoassay antigen (EIA) testing for rotavirus, adenovirus and protozoa) and compared results to multiplex real-time PCR testing. In the GEMS study, PCR increased the attributable incidence twofold for *Shigella* spp. [18]. In the MAL-ED study, PCR showed the *Shigella* burden to be more than five times higher than estimated by culture [17]. The magnitude of the difference in *Shigella* spp. yield between surveillance samples (22.7%) and routine diagnostic samples (0.8%) in our study was particularly noteworthy. *Shigella*, *Campylobacter* and *Salmonella* are included in the World Health Organisation (WHO) priority pathogens list, a list of 12 bacteria identified to pose the greatest threat to human health due to rise in antibiotic resistance [19]. The underreporting and underdiagnoses of these pathogens is therefore of public health concern.

Adenovirus, rotavirus and norovirus were detected in 25.6%, 15.6% and 14.2% of surveillance study samples respectively. These high yields are likely due in part to the significantly younger study population (mean age of 1 year) as compared to patients with samples submitted through routine diagnostic services (mean 24 years). Interviewed doctors indicated that only 34.4% would consider submitting samples for paediatric patients, likely because these are assumed to be viral infections. However, when patients under the age of 5 years were excluded from the surveillance study data, the detection rates for adenovirus (19.2%), rotavirus (12.4%) and norovirus (11.8%) remained high, indicating that these viruses are also important causes of diarrhoea in older children and adults. Less than 20% of routine diagnostic samples were submitted for adenovirus and rotavirus testing, and none for norovirus. Routine testing for viruses may be considered to be of limited benefit, as detection has little significance for clinical management, however it does have implications for antibiotic use with surveillance study data indicating that the majority of patients receiving antibiotics (60.4%) were negative for bacterial pathogens. There may also be a lack of awareness of the importance of these viral pathogens in our setting.

This study had several limitations. Due to the small sample size of the doctors’ survey, the results may not be generalizable. A more representative sample of hospitals should be drawn from a wider geographical area. This study did not investigate multifactorial associations with stool submission (a combination of patient-related factors may influence diagnostic investigation), which should be considered for future studies. Matching routine diagnostic and enhanced surveillance data sources at the patient-level was not possible as routine diagnostic data was de-identified. Despite these limitations, these data were sufficient to give an indication of trends and highlight shortfalls in routine diagnostic testing.

## Conclusion

This study showed that diarrhoeal diagnostic testing is erratic, there are inconsistencies in terms of how decisions are guided and doctors are not well informed regarding pathogens included in routine testing. Current CDW data most likely represents chronic cases, cases with dysentery and those at urban sites. Inconsistencies are recognised by doctors, who requested further training and guidance. Robust, local epidemiological data should be available to inform treatment, reserving diagnostic resources for specific cases most likely to benefit from directed rather than empiric treatment. One respondent stated that ‘if we are to practice evidence based medicine we have very little to argue for the routine diagnostic testing of stools in patients with diarrhoea. It very rarely changes clinical practice or patient outcome as it is presently practiced in our setting. Our practice has to be financially viable (feasible, sustainable), clinically meaningful, applicable and most likely in the future will be applied to a select few patients which could reap benefit from these investigations. Our studies should focus on which patients would benefit, how to identify these patients, as well as the specific therapies from which they gain improvement.’ This statement summarises the need for guidelines that are well informed by local epidemiological data thereby removing the need for testing in the majority of cases. Low pathogen yields for CDW data indicates the need for optimization of laboratory methods, including improving turnaround times and yields, for those select cases requiring diagnostics. We recommend further research into the use of molecular and antigen-based diagnostics using rectal wipes or swabs instead of whole stool samples. The increased cost of more efficient diarrhoeal diagnostics may be offset by shortened hospitalisations due to quicker resolution of illness, and savings related to decreased requirements for antibiotics. In our setting, *Shigella* spp., *Campylobacter* spp. and *Salmonella* spp. specifically are routinely under-diagnosed and require further investigation in terms of sampling, transport to the laboratory and diagnostic methods used.

## Data Availability

The datasets generated and/or analysed during the current study are not publicly available as they include study data which has not yet been published are available from the corresponding author on reasonable request.
